# The Effect of *Yucca schidigera* Extract on Serum Metabolites of Angus Crossbreed Steers with Metabolomics

**DOI:** 10.3390/metabo14010058

**Published:** 2024-01-15

**Authors:** Ziqi Deng, Baoyun Wu, Xin Yi, Jinglei Ma, Yue Liu, Luiz Gustavo Nussio, Qingxiang Meng, Zhenming Zhou, Hao Wu

**Affiliations:** 1 The State Key Laboratory of Animal Nutrition, College of Animal Science and Technology, China Agricultural University, No. 2 Yuanmingyuan West Road, Haidian District, Beijing 100193, China; s20213040607@cau.edu.cn (Z.D.);; 2 Department of Animal Science, Luiz de Queiroz College of Agriculture (Esalq), University of São Paulo, Av. Pádua Dias, 11- 13416490, Piracicaba 13418-900, SP, Brazil

**Keywords:** *Yucca schidigera* extract, Angus crossbreed steers, growth performance, nutrient digestibility, serum metabolomics

## Abstract

This study was conducted to explore the potential effect of *Yucca schidigera* extract (YSE) on the metabolism of beef cattle. Thirty Angus crossbreed steers were selected, with an initial mean body weight of 506.6 ± 33.3 kg, and assigned to two treatments: a diet with no additives (CON group) and a diet supplemented with 1.75 g/kg of YSE (YSE group) (on a dry matter basis). The experiment lasted for 104 days, with 14 days for adaptation. The results showed that adding YSE could significantly improve the average daily gain (ADG) from 1 to 59 d (15.38%) (*p* = 0.01) and 1 to 90 d (11.38%) (*p* < 0.01), as well as dry matter digestibility (DMD) (0.84%) (*p* < 0.05). The contents of alanine aminotransferase, aspartate aminotransferase, and bilirubin and the total antioxidant capacity were increased and blood urea was reduced in the YSE group, compared to the CON group (*p* < 0.05). Both the glycerophospholipids and bile acids, including phosphocholine, glycerophosphocholine, PC(15:0/18:2(9Z,12Z)), PE(18:0/20:3(5Z,8Z,11Z)), PE(18:3(6Z,9Z,12Z)/P-18:0), LysoPC(15:0), LysoPC(17:0), LysoPC(18:0), LysoPC(20:5(5Z,8Z,11Z,14Z,17Z)), deoxycholic acid, glycocholic acid, and cholic acid, were upregulated by the addition of YSE. In summary, YSE may improve the ADG by increasing the blood total antioxidant capacity and glycerophospholipid synthesis, maintaining steers under a healthy status that is beneficial for growth. Furthermore, YSE may also increase the expression of bile acid synthesis, thereby promoting DMD, which, in turn, offers more nutrients available for growth.

## 1. Introduction

Yucca plants are native to the western United States and Mexico. In traditional medicine, species of the Yucca genus have been used to treat pathologies related to inflammation, because it is rich in saponins and phenolic compounds with antioxidant effects [1]. For that reason, the dietary addition of *Yucca schidigera* extract (YSE) was initially used to reduce stress and improve the antioxidant capacity of broilers, swine, dairy cows, and steers [2,3,4,5,6]. In addition to antioxidant function, the saponins in YSE also have antiprotozoal activity [7]. Saponin is a natural cholesterol binder that can produce a stable linkage to cholesterol on the surface of the cell membrane of a protozoan so that the integrity of the cell membrane is broken, resulting in cell lysis [8]. The energy utilization in beef cattle was increased as the YSE decreased the number of protozoa, resulting in lower methane production [9,10]. Commercially, YSE, as a feed additive, is also used to control ammonia emissions and environmental impact from livestock and poultry facilities to reduce odor impact [11]. Several studies have reported that YSE could improve growth performance [5,12,13] and dry matter digestibility in ruminants [14,15]. At present, the understanding of YSE mainly focuses on its effects on rumen and intestinal microbes, rumen fermentation, and blood antioxidant properties, and the regulatory effect of YSE on animal metabolism has not been considered. The improvement in growth performance and digestibility in beef cattle is not only related to the rumen and intestinal microbes or antioxidant properties of the blood, but also directly reflected in the changes in the animal’s metabolic regulation. Thus, it is important to offer some evidence of the potential effects of YSE on the metabolism of ruminants.

Some metabolites may reflect the overall animal metabolic status and can be analyzed by untargeted metabolomics [16]. Metabolites, produced by cellular processes, can be found in the blood, as small fractions which may track the metabolic pathways. Therefore, an exploration of the blood metabolome may reveal the potential role of YSE in regulating the metabolism which is associated with growth and digestion. We hypothesize that YSE can affect the expression of certain metabolites associated with anabolism or digestion in the serum, thereby affecting growth performance and nutrient digestibility. With the aforementioned purpose, this experiment compared the growth performance, nutrient digestibility, and serum biochemical metabolome of Angus crossbreed steers receiving a basal diet or a basal diet supplemented with YSE.

## 2. Materials and Methods

### 2.1. Animal Ethics

All animal trials were approved by the Animal Welfare and Ethical Committee of China Agricultural University (Permit No. DK18030608) and performed in accordance with the Regulations for the Administration of Affairs Concerning Experimental Animals (The State Science and Technology Commission of P.R. China, 1988).

### 2.2. Diets, Animals, and Experimental Design

Thirty healthy Angus crossbreed steers with similar initial mean body weight (506.6 ± 33.3 kg) were selected and divided into two groups, using a single-factor completely randomized design. The steers were assigned to receive a basal diet with no additives in the CON group and the same basal diet supplemented with 1.75 g/kg YSE in the YSE group. The steers were fed the basal diet ad libitum (to achieve approximately 5% ort) daily, as a total mixed ration (TMR). The YSE (powder source) was premixed with 1 kg of concentrate and then mixed in a TMR wagon. YSE extracted from the whole plant of *Yucca schidigera* contains over 8% Yucca saponin (Table 1), and it was provided by Zhongnong Xingyuan Biotechnology Co., Ltd. (Beijing, China). The dosage of YSE was recommended by the manufacturers. The trial lasted for 104 d, with 14 d for adaptation, and was separated into two stages, with the respective forage-to-concentrate ratios (45:55 from 1 to 59 d and 35:65 from 60 to 90 d) to meet the nutrient requirements recommended by NASEM [17]. The diet formulas and nutritional compositions are shown in Table 2.

During the trial, individual feed intake was recorded by an automatic feed intake recording system (Shanghai Zhenghong Agricultural and Animal Husbandry Machinery Equipment Co., Ltd., Shanghai, China), body weight was determined before morning feeding monthly, and then average daily gain (ADG) was calculated. The feed conversion ratio (FCR) for each steer was determined by dividing the dry matter intake (DMI) consumed by the ADG and then presented as kg of DMI to kg of ADG. Samples of feed ingredients and TMR were collected weekly, dried for 48 h at 60 °C in a forced-air oven, and ground to pass through a 1 mm sieve for analysis of the chemical composition.

### 2.3. Feces Sampling and Processing

The feces samples were collected from the rectum of thirty steers before the morning feeding on the last day of the trial, air-dried at 65 °C, and then stored for the determination of dry matter (DM) and crude protein (CP) following the method of AOAC (2006) [18]. The apparent digestibility of dry matter (DMD) and apparent digestibility of protein (CPD) were determined using the hydrochloric acid insoluble ash method (AIA). The equation was as follows:

Apparent digestibility = 1 − (Nutrient_feces_ × AIA_diet_)/(Nutrient_diet_ × AIA_feces_) (1)

### 2.4. Serum Biochemical and Antioxidant Indices

At the end of this trial, blood samples from each steer were collected from the coccygeal vessels before the morning feeding into two 10 mL vacutainer tubes (one for blood biochemical analysis and another for serum metabolomics analysis) and then centrifuged at 4000 r/min for 15 min at 4 °C to prepare serum. Serum alanine aminotransferase (ALT), aspartate aminotransferase (AST), globulin (GLB), total protein (TP), albumin (ALB), urea, total bilirubin (TBIL), blood glucose (GLU), total cholesterol (TC), and triglycerides (TGs) were determined according to the instructions of the kit (Nanjing Jiancheng Institute of Bioengineering, Nanjing, China) using a Hitachi automatic biochemical analyzer (model 7600, Hitachi, Japan). Serum antioxidant indices, including total antioxidant capacity (T-AOC) and malondialdehyde (MDA), were determined using the colorimetric method according to the kit instructions (Nanjing Jiancheng Institute of Bioengineering, Nanjing, China).

### 2.5. LC-MS/MS-Based Untargeted Metabolomics Analysis

The LC-MS/MS analysis of samples was conducted on a Thermo UHPLC-Q Exactive HF-X system equipped with an ACQUITY HSS T3 column (100 mm × 2.1 mm i.d., 1.8 μm; Waters, Milford, MA, USA) at Majorbio Bio-Pharm Technology Co. Ltd. (Shanghai, China). The mobile phases consisted of 0.1% formic acid in water:acetonitrile (95:5, *v*/*v*) (solvent A) and 0.1% formic acid in acetonitrile:isopropanol:water (47.5:47.5, *v*/*v*) (solvent B). The flow rate was 0.40 mL/min, and the column temperature was 40 °C. MS conditions were as follows: The mass spectrometric data were collected using a Thermo UHPLC-Q Exactive HF-X Mass Spectrometer equipped with an electrospray ionization (ESI) source operating in positive mode and negative mode. The optimal conditions were set as follows: source temperature at 425 °C; sheath gas flow rate at 50 arb; Aux gas flow rate at 13 arb; ion-spray voltage floating (ISVF) at −3500 V in negative mode and 3500 V in positive mode; normalized collision energy, 20–40–60 V rolling for MS/MS. Full MS resolution was 60,000, and MS/MS resolution was 7500. Data acquisition was performed with the data-dependent acquisition (DDA) mode. The detection was carried out over a mass range of 70–1050 *m*/*z*.

### 2.6. Statistical Analysis and Annotation

Growth performance parameters, apparent digestibility, and serum indices were analyzed using SAS 9.4 and one-way analysis of variance. Differences were considered significant at *p* < 0.05 and *p* < 0.01. The data matrix obtained by searching the database was uploaded to the Majorbio cloud platform (https://cloud.majorbio.com, accessed on 21 February 2022) for data analysis. Principal component analysis (PCA) and orthogonal partial least squares discriminant analysis (OPLS-DA) were performed using the R software package “ropls” (Version1.6.2). The metabolites with VIP > 1 and *p* < 0.05 were determined as significantly different metabolites based on the variable importance in the projection (VIP) obtained using the OPLS-DA model and the *p*-value generated by Student’s *t* test. The metabolites were identified using the metabolic public databases HMDB (http://www.hmdb.ca/, accessed on 25 February 2022), Metlin (https://metlin.scripps.edu/, accessed on 25 February 2022), and Majorbio Database. The differential metabolites were annotated using the KEGG database (https://www.kegg.jp/kegg/pathway.html, accessed on 25 February 2022) to determine the pathways involved in the differential metabolites. The Python package “scipy.stats” (https://docs.scipy.org/doc/scipy/, accessed on 25 February 2022) was used to perform enrichment analysis to determine the most relevant biological pathways for experimental treatments.

## 3. Results

### 3.1. Growth Performance

As shown in Table 3, although, there was no significant difference between the treatment groups in DMI (*p* > 0.05), the dietary addition of YSE significantly increased the ADG from 1 to 59 d (*p* < 0.05) and 1 to 90 d (*p* < 0.05), with 15.38% and 11.38% more than CON group, respectively, and tended to decrease the FCR from 1 to 59 d (*p* < 0.10).

### 3.2. Nutrient Digestibility

**Figure 1:**
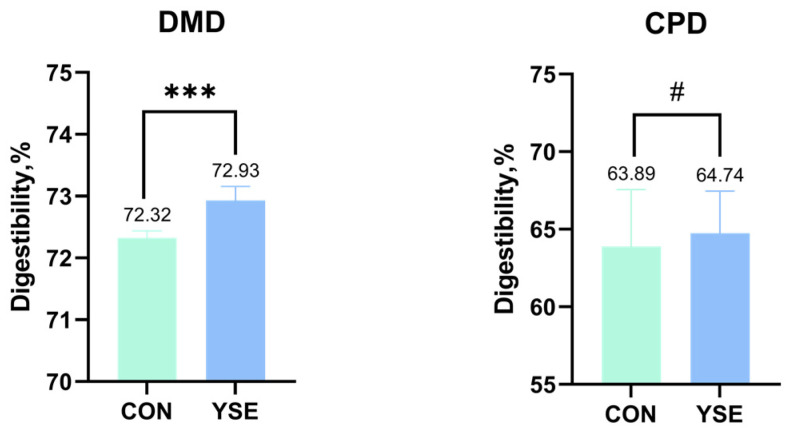
Effects of YSE on apparent nutrient digestibility of Angus crossbreed steers. Note: DMD: dry matter digestibility, CPD: crude protein digestibility. “***” indicates *p* < 0.001, “#” indicates no significance.

The apparent nutrient digestibility is presented in Figure 1. The addition of YSE significantly enhanced DMD from 72.32% to 72.93% (*p* < 0.01), which was 0.84% times higher than the control group, and had no significant effect on CPD (*p* > 0.05).

### 3.3. Serum Biochemical and Antioxidant Indices

The results of serum biochemical and antioxidant parameters are shown in Table 4. After the 90 d trial, ALT, AST, and TBIL contents were increased in the YSE group (*p* < 0.05). No significant differences in serum TP, ALB, and GLB were induced by the supplementation with YSE (*p* > 0.05). Changes in other biochemical parameters, including GLU, TC, and TGs, induced by supplementation of YSE, however, were not observed (*p* > 0.05). The serum urea molar concentration showed a significant decrease in the YSE group. As for the antioxidant indices, the T-AOC was increased with the supplementation of YSE (*p* < 0.05), even though MDA was not changed significantly (*p* > 0.05).

### 3.4. Serum Metabolites and Metabolic Pathway

#### 3.4.1. Multivariate Analysis of Serum Metabolome

**Figure 2:**
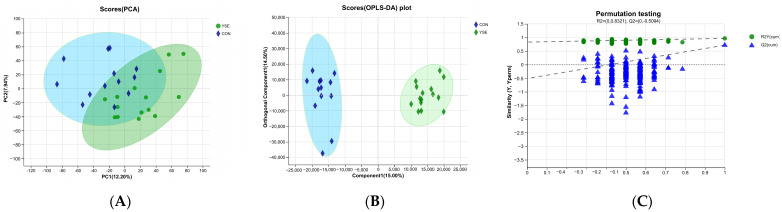
Multivariate statistical analysis of metabolites. (**A**) Unsupervised principal component analysis (PCA) of metabolites from the YSE group and CON group; (**B**) supervised orthogonal partial least squares discriminant analysis (OPLS-DA) of the metabolites from the YSE group and CON group; (**C**) permutation testing of OPLS-DA model of plot B.

Figure 2A, B reveal a relevant separation between the serum samples of the YSE and CON groups, illustrating the existence of significant differences in serum metabolites between the treatment groups. Figure 2C exhibits the reliability of the model prediction based on the robustness.

#### 3.4.2. Screening and Identification of Differential Metabolites

**Figure 3:**
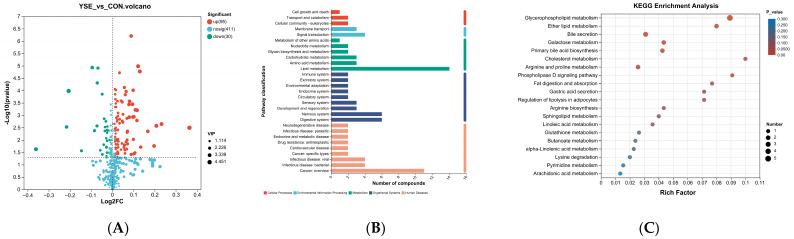
Isolation of differential metabolites and functional annotation and enrichment of KEGG. (**A**) Volcano map of blood metabolites, with VIP score of >1 and *p*-value of <0.05. (**B**) KEGG functional pathway analysis of serum differential metabolites. (**C**) Enrichment of KEGG metabolic pathways. Rich factor indicates the ratio of the number of metabolites enriched in the pathway (metabolite number) to the number of metabolites annotated to the pathway (background number).

A total of 125 named differential metabolites were found in serum samples by using LC-MS/MS analysis (VIP > 1.00, *p* < 0.05), among which 95 differential metabolites were significantly upregulated and 30 differential metabolites were significantly downregulated (Figure 3A). Then, all the differential metabolites able to be annotated by the KEGG functional pathways were classified (Figure 3B). They were related to six main categories, namely human diseases, organismal systems, metabolism, environmental information processing, and cellular processes. Consistent with the previous hypothesis, it was demonstrated that there was a close relationship with the number of compounds from the secondary metabolic pathways involved in lipid, amino acid, and carbohydrate metabolisms, as well as the overall digestive system. With the purpose of KEGG enrichment for metabolism and the digestive system, a total of 25 differential metabolic species were isolated and mainly enriched in glycerophospholipid (PL) metabolism, ether lipid metabolism, bile secretion, galactose metabolism, primary bile acid biosynthesis, and 15 other KEGG pathways, as shown in Figure 3C. The metabolites related to the top five KEGG enrichment pathways were all upregulated in the YSE group (Table 5). Nine of them were glycerophospholipids, and three of them were bile acids.

#### 3.4.3. Correlation between Serum Metabolites and Growth Performance and Digestibility

**Figure 4:**
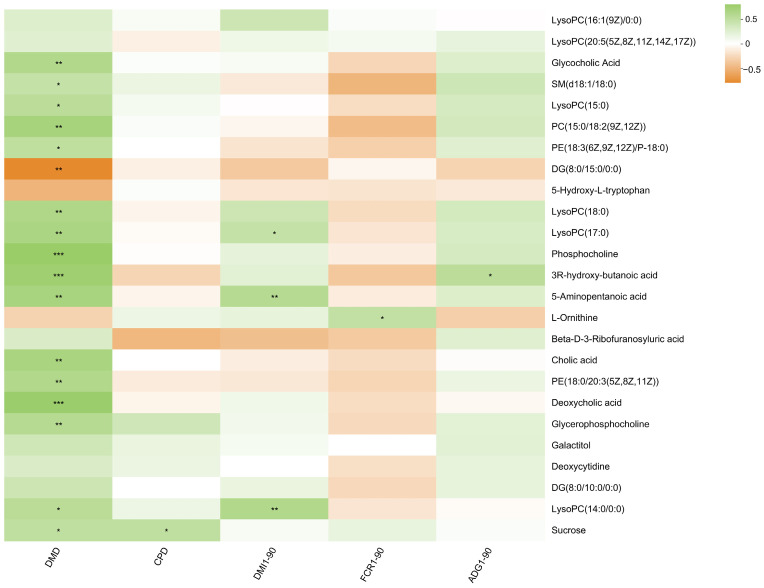
Correlation between serum metabolites, growth performance, and digestibility. “*” means a significant correlation (*p* < 0.05), “**” means a strong significant correlation (*p* < 0.01), and “***” means an extremely significant correlation (*p* < 0.001).

Through the correlation analysis of differential metabolites, growth performance, and digestibility, it was found that the digestibility of dry matter was strongly positively correlated with phospholipid metabolites and bile acid metabolites, especially phosphocholine and deoxycholic acid (*R*^2^ > 0.5, *p* < 0.001) (Figure 4).

## 4. Discussion

### 4.1. Growth Performance

The current study showed that YSE had a positive effect on the ADG, and this finding was similar to those in previous reports [5,9,10]. A higher DMI as a result of YSE supplementation was found by Abdel-Raheem [14], and the same finding was observed in this experiment. Some authors found that there were no effects of YSE in lambs on ADG, DMI, and FCR [19,20], which indicated variable effects of YSE supplementation due to the animal species, diet formulations, and YSE concentrations. Some of the main bioactive components in YSE are saponins, which have been proven to increase ADG [21]. Saponins can reduce the number of protozoa by increasing the permeability of their cell membranes, which may cause the leakage of cell contents [22]. Fewer protozoa could lead to less predation of bacteria and more microbial protein synthesis [23,24], which was supported by results showing that the YSE group had a much greater bacterial protein synthesis with a content of 19.42 μg/mL, which was increased by 49.73% compared to the CON group (unpublished data). This indicated that YSE improves ADG due to higher efficiency in the utilization of nitrogen by recycling in the rumen and increased DMD (Figure 1). It was observed that there was a reduced blood urea concentration (Table 4) as a result of the YSE supplementation, which was also in agreement with the optimized synchrony in energy and protein degradation at the ruminal level. In addition, Wallace et al. [11] documented the glycofractions of saponins binding ammonia, resulting in improved nitrogen use efficiency by decreasing the ruminal free ammonia concentration. The glycofractions of saponins trap rumen ammonia when the concentrations are at a high level after feeding, and then gradually release ammonia for microbial protein synthesis over time and according to the ruminal ammonia level decay [21]. Moreover, supplementing a poultry diet with YSE was reported to improve carcass weight and meat quality, including pH, drip loss, and lightness [25], and YSE supplementation was also reported to result in a higher lamb carcass weight [19]. With regard to meat chemical composition, Benamirouche [26] highlighted the increase in protein and decrease in lipid contents in the breast and thigh muscles in broilers. However, those effects remain unknown in beef cattle. Therefore, the effect of YSE on the carcass parameters and meat quality of beef cattle needs to be further explored.

### 4.2. Nutrient Digestibility

The nutrient digestibility of ruminants is largely dependent on ruminal efficiency, as microorganisms degrade and utilize the majority of NDF, starch, nitrogen-free extract, and CP. Previous studies [11,24,27,28] have shown that YSE increased DM, starch, and NDF digestibility and decreased CP digestibility by regulating the rumen microflora and altering the activity of the CMCase enzyme and amylase enzyme in the rumen. However, Anele et al. [29] found no effects on in vitro dry matter disappearance. The reasons for the inconsistent results may be due to the large difference between the in vitro and in vivo environments and the forage:concentrate ratio in the tested diets. Abdel-Raheem [14] mentioned that YSE improved dry matter, ether extract, and nitrogen-free extract digestibility because of its saponin content which affected the biosynthesis of rumen microorganisms. Furthermore, Yi et al. [30] found that YSE reduced the relative abundance of *Bacillota* and increased the relative abundance of *Bacteroidota*. *Bacteroidota* was mainly involved in the degradation of non-fibrous polysaccharides, while *Bacillus* was mainly involved in the catabolism of fibrous polysaccharides [31,32]. After cellulose is broken down by the cellulase produced by *Bacillota*, then *Bacteroidota* can efficiently use glucans, fructans, xylans, and arabinans because they have more genes encoding glycoside hydrolase and polysaccharide lyase [33]. Moreover, YSE has shown effects on digestive and absorption capacity at the intestinal level. Mao et al. [34] reported that the addition of YSE in laying hens’ diets mitigated the negative effect of *Clostridium perfringens* and coccidia infection on morphology, functions, cell apoptosis, and antioxidant capacity in the jejunum. Yang et al. [35] found that YSE promoted villus growth and increased villus height/crypt depth in piglets.

### 4.3. Serum Biochemical and Antioxidant Indices

Serum biochemical indicators reflect the function and physiological state of body tissues and organs, as well as the metabolism of nutrients and the status of the immune system. Serum ALT and AST are two important transaminases that not only accelerate protein synthesis and metabolism and improve the body’s conversion efficiency of nutrients but also reflect whether the liver is functioning normally [36]. An abnormal increase in AST and ALT activity indicates that liver function is reduced due to liver injury [37]. In this experiment, even though the activities of AST and ALT in the YSE group were significantly higher than those in the CON group, they still remained within the reference range (12.9~104.2 U/L and 3.8~41.4 U/L) for beef cattle [38]. TBIL is a metabolite of heme in red blood cells and also a natural antioxidant that scavenges oxygen free radicals and improves the body’s ability to resist stress. When serum bilirubin is too high, it usually predicts abnormal liver lesions or bile duct obstruction. In the present study, TBIL in the YSE group was higher than that in the CON group, but within the normal reference range, which indicated that the addition of YSE did not damage liver function. As shown in Table 4, the TP, ALB, GLB, and urea levels jointly reflected the body’s metabolism and utilization of protein. Serum TP is the sum of ALB and GLB, and its content is positively correlated with protein synthesis and metabolism in body tissues [39]. ALB is a mediator of anabolism in animal tissues, and serum GLB is closely related to animal immunity [26]. The results of this current study are in accordance with those of Chen [40], who observed no beneficial effect of YSE supplementation in sow diets on TP. However, we observed a significant decrease in blood urea, in agreement with other studies [21], due to Yucca extracts being rich in resveratrol and saponins. Both compounds are urease inhibitors [41] and may reduce rumen ammonia production, resulting in a decrease in urea entering the blood. Resveratrol is a molecule with antioxidant properties that can exert the ability to either directly neutralize reactive oxygen species or indirectly upregulate the expression of cellular defensive pathways and genes [42]. A previous study showed that YSE increased the levels of blood GLU and TGs and reduced TC levels [43]; however, these effects were not noticed in the current study. T-AOC reflects the total antioxidant capacity of some antioxidant molecules and enzymes. MDA is the main product of lipid peroxidation and a biomarker used to determine whether an organism is in a state of oxidative stress [44]. In the current study, YSE was found to have the effect of increasing the antioxidant capacity of beef steers, which was probably due to the presence of resveratrol and other polyphenols in YSE [45].

### 4.4. Serum Metabolites and Metabolic Pathway

**Figure 5:**
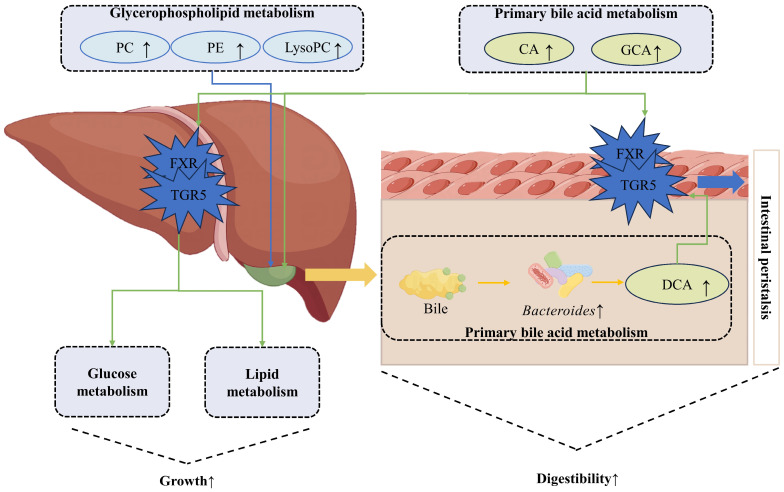
The metabolic mechanisms of YSE affecting growth and digestibility in Angus crossbred steers. PC: phosphatidylcholine; PE: phosphatidylethanolamine; LysoPC: lysophospholipid; CA: cholic acid; GCA: glycocholic acid; DCA: deoxycholic acid. FXR and TGR5 are two bile acid receptors.

Small-molecule metabolites represent the end products of the body’s regulatory processes, and sudden changes in protein and gene expression may reflect the status of metabolic challenge. To the best of our knowledge, this is the first time it has been proposed that YSE might increase ADG and DMD through upregulating glycerophospholipid metabolism and bile acid metabolism (Figure 5).

#### 4.4.1. Glycerophospholipid Metabolism

Glycerophospholipid (PL) is the most abundant type of phospholipid in the body. According to the different substituents, it can be divided into a few categories, mainly phosphatidylcholine (PC), phosphatidylethanolamine (PE), phosphatidylserine, and phosphatidylinositol. Lysophospholipid (LysoPC) is a metabolite of PC, and phosphocholine is a precursor to the synthesis of phosphatidylcholine [46]. In this trial, the phosphocholine content of the YSE group was increased, which resulted in enhanced synthesis of PC. At the same time, PE might also be converted to PC under the action of phosphatidylethanolamine N-methyltransferase. Phospholipases A1 and A2 could, then, accelerate PC degradation and release free fatty acids and LysoPC. PLs are involved in many vital activities of the body, including cellular integrity, proliferation, differentiation, and immunity [47,48]. Antigen recognition by T lymphocytes requires the involvement of antigen-presenting cells. When phospholipids on the membrane of antigen-presenting cells are disrupted, the binding of antigens to antigen-presenting cells (APCs) is blocked [49]. Phosphatidylcholine and lysolecithin are associated with the binding of APCs and antigens [49]. Based on those observations, it might be suggested that YSE could improve animal immunity by increasing the content of glycerophospholipids in the serum, enhancing the binding ability of cell membranes. Moreover, it is generally accepted that glycerophospholipid metabolism plays a vital role in maintaining liver metabolism. The dysregulation of phospholipid metabolism in the process of glycerophospholipid metabolism is related to liver diseases such as steatosis and fibrosis [50]. Regulating phospholipid metabolism could reduce phospholipid disorders, which is beneficial for liver protection and treatment [51]. In addition, acute liver injury can cause hepatocyte membranes to be damaged and disrupted, resulting in an imbalance in phosphatidylcholine and phosphatidylethanolamine metabolism and ultimately a decrease in lysolecithin content [52]. In addition, YSE promotes the synthesis of LysoPC (20:5(5Z,8Z,11Z,14Z,17Z)), LysoPC (15:0), LysoPC (17:0), and LysoPC (18:0) in the blood, which is conducive to maintaining the balance of glycerophospholipid metabolism, reduces cell damage and inflammation, and ultimately plays a protective role in the liver. More importantly, PC is the main component of bile with biological functions, like promoting bile synthesis and secretion, enhancing the emulsification of fat, and participating in the enterohepatic circulation of bile acids [53].

#### 4.4.2. Bile Acid Metabolism

Bile acids can be divided into primary and secondary bile acids according to their synthetic source. The synthesis of primary bile acid from cholesterol, including cholic acid (CA) and chenodeoxycholic acid, occurs in liver cells. Then, primary bile acids combined with taurine or glycine will form taurocholic acid and glycocholic acid (GCA), respectively, which are stored in the gallbladder as bile salts. After being secreted into the duodenum through the bile ducts, primary bile acids are modified by enzymes produced by bacteria through removal, oxidation, or epimerization of the unclear hydroxyl groups to form secondary bile acids, including deoxycholic acid (DCA) and lithocholic acid [54]. In this trial, YSE increased the content of CA, GCA, and DCA in the serum of steers, which might be related to the previously mentioned effects of enhanced glycerophospholipid content, namely maintaining liver health and promoting bile acid reabsorption. Li et al. [55] reported that *Bacteroides* were associated with secondary bile acid production, and Yi et al. mentioned an increased abundance of *Bacteroides* with YSE supplementation. This resulted in an increase in the level of secondary bile acids in the serum (Table 5). Bile acids and PC can participate in the emulsification of fat and promote fat digestion and absorption in the duodenum [56,57]. Bile acids also bind to dietary proteins in the small intestine during digestion. More rapid proteolysis by pancreatic proteases is promoted by this binding effect [58]. Furthermore, bile acids can stimulate intestinal peristalsis and facilitate digestion of the diet in the intestine [59]. Through blood transport, bile acids can produce signal regulation effects on tissues and organs. FXR and TGR5 are two bile acid receptors that are widely found in various organs and tissues in the body. Bile acids can maintain lipid homeostasis and glucose homeostasis by activating these receptors [60]. In the current study, both primary and secondary bile acids showed an increase, and the relatively stable levels of GLU, TC, and TGs in the blood may be due to the regulatory effect of bile acids.

## 5. Conclusions

In summary, this study demonstrated that the application of YSE as a natural growth promoter has useful effects on the growth performance, digestibility, and total antioxidant capacity of Angus crossbreed steers. In addition, YSE could also alter the expression of 125 metabolites in the blood of steers, including glycerophospholipids and bile acids. The increased levels of glycerophospholipids and bile acids may explain, at least in part, the beneficial effects on the growth performance and nutrient digestibility of Angus crossbreed steers.

## Figures and Tables

**Table 1:** Composition of *Yucca schidigera* extract (air-dry basis).

Item	Content
*Yucca schidigera* powder	≥30.0%
Total saponins	≥8.0%
Moisture	≤7.0%
Ash	<10.0%

**Table 2:** Diet formula and nutritional composition of the basal diets (DM basis).

Item	Day_1–59_	Day_60–90_
Ingredients, %		
Corn silage	24.75	19.25
Corn stalk	20.25	15.75
Soybean meal	9.35	11.05
Jujube powder	8.25	9.75
Ground corn	31.90	37.70
Salt	1.10	1.30
Premix 1	2.20	2.60
Calcium hydrophosphate	1.10	1.30
Sodium bicarbonate	1.10	1.30
Nutritional composition		
Metabolizable energy, MJ/kg	10.42	10.59
Crude protein, %	11.33	11.94
Neutral detergent fiber, %	41.75	33.84
Acid detergent fiber, %	16.20	13.89
Ether extract, %	2.82	2.83
Starch, %	26.47	31.90
Calcium, %	0.50	0.52
Total phosphorus, %	0.43	0.48

1 Premix (per kg of DM) contains 150,000–450,000 IU vitamin A acetate, 40,000–120,000 IU vitamin D3, 400 mg DL-α-tocopherol acetate, 250–750 mg copper, 1000–5000 mg iron, 1000–3000 mg manganese, 1500–3700 mg zinc, 10–25% calcium, 0.3% total phosphorus, and 15–30% sodium chloride. 2 ME (metabolizable energy) was calculated, and other components were determined based on NASEM (2016) [17].

**Table 3:** Effects of YSE on growth performance of Angus crossbreed steers.

Item	Treatments		SEM	*p*-Value
	CON	YSE		
Body weight (kg)				
Day_1_	504.93	508.63	6.09	0.77
Day_60_	573.53	588.37	6.69	0.28
Day_90_	611.80	630.70	6.67	0.16
ADG (kg/d)				
Day_1–59_	1.17	1.35	0.03	0.01
Day_60–90_	1.23	1.37	0.07	0.35
Day_1–90_	1.19	1.36	0.03	<0.01
DMI (kg/d)				
Day_1–59_	12.41	12.60	0.21	0.67
Day_60–90_	11.89	12.52	0.24	0.20
Day_1–90_	12.21	12.55	0.21	0.42
FCR				
Day_1–59_	10.16	9.34	0.25	0.09
Day_60–90_	9.60	9.00	0.41	0.48
Day_1–90_	9.82	9.29	0.19	0.16

Note: CON: control group; YSE: *Yucca schidigera* extract group; ADG: average daily gain; DMI: dry matter intake; FCR: feed conversion ratio (DMI/ADG).

**Table 4:** Effects of CAP and YSE on serum biochemical and antioxidant indices of Angus crossbreed steers.

Item	Treatments		SEM	*p*-Value
	CON	YSE		
ALT (U/L)	34.34	40.92	1.09	<0.01
AST (U/L)	76.55	84.66	1.26	<0.01
TBIL (μmol/L)	1.83	1.97	0.03	0.02
TP (g/L)	69.95	71.18	0.61	0.32
ALB (g/L)	37.34	37.07	0.31	0.68
GLB (g/L)	32.61	34.11	0.55	0.17
Urea (mmol/L)	3.53	3.31	0.05	0.03
GLU (mmol/L)	4.95	5.07	0.05	0.29
TC (mmol/L)	2.96	3.02	0.37	0.48
TGs (mmol/L)	0.25	0.25	0.14	0.29
T-AOC (U/mL)	0.23	0.24	0.00	0.02
MDA (nmol/mL)	3.93	3.83	0.21	0.38

Note: ALT: alanine aminotransferase; AST: aspartate aminotransferase; GLB: globulin; TP: total protein; ALB: albumin; TBIL: total bilirubin; GLU: blood glucose; TC: total cholesterol; TGs: triglycerides; T-AOC: total antioxidant capacity; MDA: malondialdehyde.

**Table 5:** Differential metabolites enriched in the top five KEGG pathways related to metabolism and the digestive system.

Metabolite	Formula	RT (min)	M/Z	*p*-Value	Regulate
Phosphocholine	C_5_H_14_NO_4_P	6.188	184.0732	<0.001	up
Glycerophosphocholine	C_8_H_20_NO_6_P	0.459	280.0918	<0.001	up
PC(15:0/18:2(9Z,12Z))	C_41_H_78_NO_8_P	6.168	788.5465	0.018	up
PE(18:0/20:3(5Z,8Z,11Z))	C_43_H_80_NO_8_P	6.182	814.561	0.014	up
PE(18:3(6Z,9Z,12Z)/P-18:0)	C_41_H_76_NO_7_P	6.084	770.5361	0.045	up
LysoPC(15:0)	C_23_H_48_NO_7_P	5.951	480.3106	0.017	up
LysoPC(17:0)	C_25_H_52_NO_7_P	6.834	508.3414	<0.001	up
LysoPC(18:0)	C_26_H_54_NO_7_P	6.841	568.3627	<0.001	up
LysoPC(20:5(5Z,8Z,11Z,14Z,17Z))	C_28_H_48_NO_7_P	6.018	586.3161	0.020	up
Cholic acid	C_24_H_40_O_5_	5.965	407.2808	<0.001	up
Glycocholic acid	C_26_H_43_NO_6_	6.231	464.302	<0.001	up
Deoxycholic acid	C_24_H_40_O_4_	6.018	391.2861	0.040	up
Galactitol	C_6_H_14_O_6_	0.697	205.068	0.023	up

Note: PC: phosphatidylcholine; PE: phosphatidylethanolamine; LysoPC: lysophospholipid.

## Data Availability

Data presented in this study are available on request from the corresponding author. Data are available on request due to ethical restrictions.

## Abbreviations

The abbreviations in this article are as follows:

**Abbreviation**	**Meaning**
ADG	average daily gain
ALB	albumin
ALT	alanine aminotransferase
AST	aspartate aminotransferase
CA	cholic acid
CPD	apparent digestibility of crude protein
DCA	deoxycholic acid
DMD	apparent digestibility of dry matter
DMI	dry matter intake
FCR	feed conversion ratio
GCA	glycocholic acid
GLB	globulin
GLU	blood glucose
LysoPC	lysophospholipid
MDA	malondialdehyde
PC	phosphatidylcholine
PE	phosphatidylethanolamine
T-AOC	total antioxidant capacity
TBIL	total bilirubin
TC	total cholesterol
TGs	triglycerides
TMR	total mixed ration
TP	total protein
YSE	*Yucca schidigera* extract
